# Functional Magnetic Resonance Imaging of Electrical and Optogenetic Deep Brain Stimulation at the Rat Nucleus Accumbens

**DOI:** 10.1038/srep31613

**Published:** 2016-09-07

**Authors:** Daniel L. Albaugh, Andrew Salzwedel, Nathalie Van Den Berge, Wei Gao, Garret D. Stuber, Yen-Yu Ian Shih

**Affiliations:** 1 Department of Neurology, University of North Carolina, Chapel Hill, NC, 27599, USA; 2 Biomedical Research Imaging Center, University of North Carolina, Chapel Hill, NC, 27599, USA; 3 Curriculum in Neurobiology, University of North Carolina, Chapel Hill, NC, 27599, USA; 4 Department of Radiology, University of North Carolina, Chapel Hill, NC, 27599, USA; 5 Biomedical Imaging Research Institute, Department of Biomedical Sciences and Imaging, Cedars-Sinai Medical Center, Los Angeles, CA, 90048, USA; 6 Medical Image and Signal Processing Group, Ghent University, Ghent, 9000, Belgium; 7 Department of Psychiatry, University of North Carolina, Chapel Hill, NC, 27599, USA; 8 Department of Biomedical Engineering, University of North Carolina, Chapel Hill, NC, 27599, USA

## Abstract

Deep brain stimulation of the nucleus accumbens (NAc-DBS) is an emerging therapy for diverse, refractory neuropsychiatric diseases. Although DBS therapy is broadly hypothesized to work through large-scale neural modulation, little is known regarding the neural circuits and networks affected by NAc-DBS. Using a healthy, sedated rat model of NAc-DBS, we employed both evoked- and functional connectivity (fc) MRI to examine the functional circuit and network changes achieved by electrical NAc stimulation. Optogenetic-fMRI experiments were also undertaken to evaluate the circuit modulation profile achieved by selective stimulation of NAc neurons. NAc-DBS directly modulated neural activity within prefrontal cortex and a large number of subcortical limbic areas (e.g., amygdala, lateral hypothalamus), and influenced functional connectivity among sensorimotor, executive, and limbic networks. The pattern and extent of circuit modulation measured by evoked-fMRI was relatively insensitive to DBS frequency. Optogenetic stimulation of NAc cell bodies induced a positive fMRI signal in the NAc, but no other detectable downstream responses, indicating that therapeutic NAc-DBS might exert its effect through antidromic stimulation. Our study provides a comprehensive mapping of circuit and network-level neuromodulation by NAc-DBS, which should facilitate our developing understanding of its therapeutic mechanisms of action.

Therapeutic deep brain stimulation (DBS) therapy has become an increasingly important clinical tool in neuropsychiatry^1^,^2^. Among several targets under active clinical consideration for neuropsychiatric DBS, the nucleus accumbens (NAc, ventral striatum) is perhaps the most common. Functionally interfaced with both limbic and executive circuitry^3^, the NAc has emerged as a promising target for several neuropsychiatric conditions, including obsessive-compulsive disorder (OCD)^4^ and treatment-resistant depression^5^.

A longstanding issue with DBS therapy is the unpredictability of the cellular, circuit- and network-level responses to site-specific electrical stimulation, particularly at the high stimulation frequencies that are commonly employed (>100 Hz)^6^. Indeed, despite active clinical use, the therapeutic mechanisms of NAc-DBS are poorly understood, impeding the development and refinement of this promising therapy. Functional brain mapping tools, including positron emission tomography (PET) and functional magnetic resonance imaging (fMRI), are promising avenues for the study of circuit and network modulation by DBS therapy^7^,^8^. The key strengths to fMRI-based studies of DBS include the ability to detect dynamic changes in stimulus-evoked activity and connectivity, *in vivo*, across the whole brain with reasonably high spatiotemporal resolution. Unfortunately, the implementation of fMRI tools to study NAc-DBS in clinical populations is strongly limited by both obstructive electrode imaging artifacts and appropriate safety considerations (largely pertaining to dangers associated with the introduction of DBS equipment to the MR environment)^9^.

In contrast to clinical populations, animal models of DBS can be readily studied with fMRI under far more flexible experimental conditions^10^,^11^,^12^,^13^. For example, long-duration scan sessions can be conducted to map the acute neural responses to DBS at varying stimulation frequencies. This is particularly important as DBS therapy for both movement and neuropsychiatric disorders is commonly employed only at high stimulation frequencies, suggesting frequency-dependent mechanism(s) of action^6^. In the case of NAc-DBS, the effects of stimulation frequency on neural circuit modulation have not been extensively explored using neuroimaging tools. DBS-fMRI in animal models also offers flexibility in electrode choice, in particular those built using more MR-compatible materials, reducing obstructive imaging artifacts^14^.

In the present study, we employed simultaneous DBS and multimodal fMRI to study the circuit and network-level effects of NAc stimulation in a healthy rat model. This model was chosen based on its widespread use in preclinical studies of NAc-DBS^15^,^16^,^17^,^18^,^19^,^20^. Electrical stimulation of the NAc strongly modulated prefrontal cortex and a diverse complement of subcortical limbic regions, including many areas not previously detected by DBS-fMRI. We also provide the first global functional connectivity mapping of NAc-DBS at therapeutic stimulation parameters. Broadly, these DBS-fMRI measurements provide critical information regarding both the pathways modulated by NAc-DBS, as well as the strength of modulation by DBS at therapeutic parameters. Interestingly, the detected pattern and size of CBV modulation by NAc DBS was largely insensitive to stimulation frequency. Supplementing our electrical DBS findings, we describe exploratory optogenetic-fMRI experiments with selective stimulation of NAc neurons.

## Materials and Methods

### Subjects

A total of 15 male Sprague-Dawley rats (300–500 g, Charles River Laboratories, Wilmington MA) were included in this study. Rats were individually housed in cages with food and water available ad libitum and 12:12 day–night cycles with control of humidity and temperature. All procedures were performed in accordance with the National Institutes of Health Guidelines for Animal Research (Guide for the Care and Use of Laboratory Animals) and approved by the University of North Carolina Institutional Animal Care and Use Committee.

### Experimental Overview

This study broadly consisted of four different experiments: 1) Evoked-fMRI of electrical NAc-DBS at 130 Hz (300 μA stimulation); 2) Evoked-fMRI of electrical NAc-DBS to examine frequency-dependency of fMRI responses (10, 40, 70, 130, 200 Hz; 500 μA stimulation); 3) Functional connectivity (fc) MRI measurements of network-level responses to NAc-DBS at 130 Hz (300 μA stimulation); and 4) Evoked-fMRI responses to selective optogenetic stimulation of NAc neurons. Evoked- and fcMRI experiments were conducted in the same group of subjects (and scanning session), whereas optogenetic experiments were conducted in a separate group of subjects.

### Stereotactic Surgery

For all surgical procedures, rats were endotracheally intubated and mechanically ventilated using a small animal ventilator (CWE Inc., SAR-830/PA, Ardmore, PA). Anesthesia was maintained under a constant flow of 2% isoflurane mixed with medical air, and physiological parameters were continuously monitored and maintained within normal ranges using capnometry (Surgivet, Smith Medical, Waukesha, WI) and pulse oximetry (MouseOx Plus, STARR Life Science Corp., Oakmont, PA). Animals were head-fixed to a stereotactic frame (Kopf Instruments, Model 962, Tujunga, CA), and burr holes were prepared according to experimental coordinates. For electrical DBS experiments, homemade MRI-compatible two-channel tungsten microelectrodes were fabricated as previously described^14^, and targeted to the mediodorsal boundary of the NAc core/shell (2.28 mm anterior to bregma, 1.2 mm right of midline, and 6.6 mm ventral to cortical surface). Two electrode leads (50 μm each in diameter, approximately 1 cm in length), were adhered with a saturated sucrose solution for minimal interspace distance. These electrodes were fully insulated with polyimide except at the tips, with an *in vitro* impedance of 18–22 kΩ at 1 kHz^14^. For optogenetic DBS experiments, viral microinjections were targeted to the same stereotactic coordinates used for electrode implantations. Injections were administered as either 1 or 2 μl volumes at a flow rate of 0.1 and 0.2 μl/min, respectively (total infusion time = 10 min). An additional 10 min was given for virus diffusion prior to slow retraction of the microsyringe needle. Chronically-implantable optical fibers (200 μM; NA: 0.37)^21^ were placed 0.5 mm above virus injection sites.

For all experiments, MR-compatible miniature brass screws (Item #94070A031, McMaster Carr, Atlanta, GA) were anchored to the skull, and dental cement was used to seal implanted components. Surgical sutures were employed to further protect the surgical site. For electrical DBS experiments, a recovery period of at least 24 h was given prior to fMRI, while at least three weeks were given for optogenetics experiments to allow for sufficient viral expression.

### Functional MRI Scan Preparation

In preparation for fMRI procedures, rats were endotracheally intubated and mechanically ventilated using a small animal MR-compatible ventilator (CWE Inc., MRI-1, Ardmore, PA). Anesthesia was initially maintained under constant isoflurane (1.5–2%) mixed with medical air. Next, tail vein catheterization was performed for intravenous drug and contrast agent injections (see below). Immediately following intubation and tail vein catheterization, animals were placed within a head-holder, and harnessed to a small animal cradle (both plastic and custom-made). The cradle was lined with a circulating water blanket connected to a temperature-adjustable water bath located outside the scanner room (Thermo Scientific, Waltham, MA). A rectal probe was employed and core body temperature was maintained at 37 ± 0.5 °C. Mechanical ventilation volume and rate were adjusted to maintain EtCO_2_ of 2.8–3.2% and SpO_2_ above 96%, using capnometry (Surgivet, Smith Medical, Waukesha, WI) and pulse oximetry (MouseOx Plus, STARR Life Science Corp., Oakmont, PA). EtCO_2_ values from the capnometry system were previously calibrated against invasive sampling of arterial blood gas, reflecting a pCO_2_ level of 30–40 mm Hg^22^,^23^.

### Functional MRI

**Figure 1:**
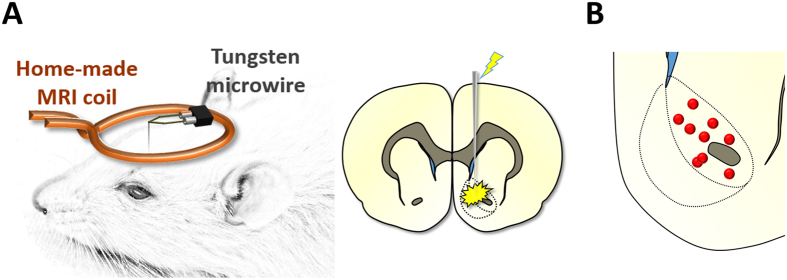
**(A)** Schematic of experimental imaging setup with a custom single loop surface coil and a tungsten microwire electrode. **(B)** Electrode tip mapping to the NAc for all electrical DBS subjects (*n* = 9). Tip placements were estimated using T_2_-weighted anatomical scans, which we deemed satisfactory given the relatively large size of the NAc (including anteroposterior distance), as well as the reduced electrode artifact.

MR images were acquired on a 9.4-Tesla Bruker BioSpec system with a BGA-9S gradient insert (Bruker Corp., Billerica, MA). A homemade single-loop surface coil with an internal diameter of 1.6 cm, placed directly over the head, was used as a transceiver. Toothpaste was applied within the open coil loop to minimize MR susceptibility artifacts. The set-up of the coil and DBS electrode is shown in Fig. 1A.

Magnetic field homogeneity was optimized first by global shim and followed by local first- and second-order shims using the FASTMAP protocol^24^. For anatomical referencing, a T_2_-weighted RARE pilot image was taken in the mid-sagittal plane to localize the anterior commissure; this structure is located at approximately 0.36 mm posterior to the bregma and served as a reference for anteroposterior slice positioning in subsequent anatomical and functional scans. T_2_-weighted anatomical images were obtained using a RARE sequence (scan parameters: TR = 2500 ms, TE_eff_ = 33 ms, RARE factor = 8, slice thickness = 1 mm, matrix size = 256 × 256, FOV = 2.56 × 2.56 cm^2^). Twelve coronal slices were acquired, with the 5th slice from the anterior direction aligned with the anterior commissure (as revealed in the previous T_2_-weighted pilot scan). The reduced electrode distortion artifact (Supplemental Figure S1), together with standardized slice positioning, rendered these images sufficient to localize the electrode tip placement, as previously described^12^,^14^. As visualized slices were 1 mm thick, all correctly targeted electrodes fell within this coronal section we deemed this resolution adequate given the relatively large anteroposterior size of the NAc. For optogenetic-fMRI experiments, optical fiber placements above the NAc virus injection site were similarly visualized using the anatomical images. Electrode and optical fiber placements within the NAc were confirmed for each rat (Fig. 1B and Supplemental Figure S1); animals with placements outside of the target regions or defective electrodes were discarded for subsequent experiments (*n* = 3).

Following setup processes and immediately prior to cerebral blood volume (CBV) fMRI scan acquisition, rats were administered Feraheme (30 mg Fe/kg, i.v.). Subsequently, anesthesia was switched from 1.5–2% isoflurane to sedation using dexmedetomidine (dexdomitor; 0.05 mg/kg/hr, i.v.) cocktailed with the paralytic agent pancuronium bromide (0.5 mg/kg/hr, i.v.) (to facilitate mechanical ventilation). This cocktail was administered for the remaining scan duration, continuously supplemented by 0.5% isoflurane^25^. A delay of at least 10 min was given before beginning fMRI experiments, to allow animals to adjust to sedation. Total experimental duration was limited to a maximum of three hours (following dexmedetomidine administration for all subjects.

CBV fMRI was chosen against the more traditional blood-oxygen-level-dependent (BOLD) fMRI due to its superior contrast-to-noise ratio^26^. CBV fMRI scans were acquired using a multi-slice single-shot gradient echo echo-planar imaging sequence (GE-EPI) (scan parameters: TR = 1000 ms, TE = 8.1 ms, bandwidth = 250 kHz, slice thickness = 1 mm, matrix size = 80 × 80 (zero-padding to 128 × 128), and FOV = 2.56 × 2.56 cm^2^. Image slice geometry was imported from the previously acquired T_2_-weighted anatomical image (12 slices).

### Deep Brain Stimulation (DBS)

Simultaneous electrical DBS with CBV fMRI was acquired in the same manner for all subjects showing accurate electrode placement (*n* = 9), using bipolar and uniformly-distributed unilateral stimulation of the right NAc. Subjects with inaccurate electrode placement were excluded for subsequent experiments. Each stimulation period consisted of a series of TTL-triggered biphasic, charge-balanced square-wave pulses delivered at 130 Hz, with a stimulation intensity of 300 μA and pulse duration of 90 μs (Experiment 1). A 70 s block design paradigm was implemented (adopted from one of our previous DBS-fMRI studies)^12^, consisting of 20 s of rest (stimulation OFF) followed by 10 s of stimulation ON, and an additional 40 s of rest (stimulation OFF). In a second set of experiments designed to test frequency-dependency of DBS-evoked CBV modulation (Experiment 2), higher current amplitude was employed (500 μA). These experiments, conducted prior to the observation of detectable CBV responses at 300 μA, nevertheless fall within normal amplitude ranges (or lower) for rodent DBS-fMRI experiments^12^,^27^. Stimulation frequencies were varied in a pseudo-randomized order (i.e., 130, 40, 70, 200, 10 Hz), consistent across subjects yet designed to eliminate potential stimulation frequency order effects. The application of a fixed pulse duration in this experiment (i.e., 90 μs), while varying total charge delivered by stimulation frequency, was chosen to mimic the short pulse durations used clinically, which may be biased for targeting axons over somata^28^. Each DBS frequency scan was repeated 5-times per rat for within-subject/session averaging (note that all descriptions of *n* refer to total rat number). An additional rest period of at least two min was given between all evoked-fMRI DBS scans (electrical and optogenetic [see below]) to allow for neurovascular recovery.

For Experiment 3, fcMRI scans were acquired using EPI scan parameters identical to those for evoked-fMRI data (see above), however with a 5 min (300 frame) scan duration (*n* = 7). First, a pre-stimulation (“resting”) baseline scan was acquired, wherein no NAc-DBS was applied. Immediately afterwards, a second fcMRI scan was acquired with simultaneous, continuous 130 Hz NAc-DBS (300 μA). This was again followed by a resting, post-stimulation scan in which no DBS was applied.

In Experiment 4, a pilot experiment examining the evoked-fMRI responses to optogenetic stimulation at NAc (*n* = 4 for ChR2; *n* = 2 for EYFP control), a 473 nm wavelength diode-pumped solid-state (DPSS) laser (model BL473T8-200, Shanghai Laser & Optics Century, Shanghai, China) was connected via coupler to a homemade patch cable terminating above the chronically-implanted optical fibers. Wavelength-specific light output at the terminating end of the patch cable was pre-calibrated to 20 mW using a wattage meter. Optogenetic stimulation periods consisted of a series of TTL-triggered light pulses with a stimulation frequency of 40 Hz and pulse duration of 5 or 10 ms. A 100 s block design paradigm was implemented, consisting of 20 s rest (stimulation OFF) followed by two 10 s stimulation periods (stimulation ON), with intervening and final rest periods of 30 s.

### Stimulus-Evoked Data Processing and Statistical Analyses

Preprocessing and image analysis was performed using SPM codes and a custom-written program in Matlab (MathWorks Inc., Natick, MA) similar to our previous DBS-fMRI studies^10^,^12^,^14^. CBV fMRI data were automatically realigned to the first volume of a well-positioned subject. These datasets were semi-automatically skull-stripped using a thresholding method and coregistered to an anatomical MRI rat atlas^29^.

Functional DBS response maps were generated using the general linear model (GLM), statistical significance was set at *p* ≤ 0.05. To control for multiple comparisons, the Benjamini and Hochberg/Yekutieli procedure for controlling false discovery rate was utilized^30^. All images were smoothed by applying a mean filter with a 3 × 3 kernel, and overlaid on an anatomical MRI rat atlas^29^. Responses are expressed in T-score units ranging from 2 to 10.

**Figure 2:**
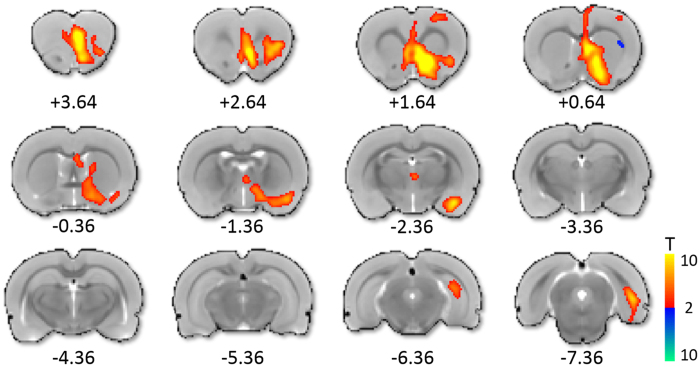
Functional CBV activation maps by 130 Hz NAc-DBS (300 μA; *n* = 5). CBV modulation was largely ipsilateral to the stimulated hemisphere, was predominantly positive in direction, and included both cortical and subcortical CBV modulation. Notable regions demonstrating CBV increases included the prefrontal cortex (including prelimbic, infralimbic, and orbitofrontal), NAc, lateral hypothalamus, amygdala, ventral hippocampus and others. CBV decreases were also detected within a small region of the ipsilateral dorsal striatum. 12 slices were acquired in each scan, with numbers below slices denoting relative distance from bregma (in mm). Color bar denotes t score values obtained by GLM analyses, with a significance threshold of *p* < 0.05 (corrected). Functional activation maps for all additional tested frequencies are located in Supplemental Figures S4.

For temporal analysis of electrical DBS-evoked CBV changes, 3-dimensional regions of interest (ROIs) were defined *a priori* according to anatomical structural boundaries^29^,^31^, and applied onto the coregistered data. The ROIs chosen for time-course analysis included only those regions showing statistically significant modulation during 130 Hz DBS with 300 μA current (see Fig. 2).

The effect of DBS stimulation frequency on ΔCBV change was calculated by averaging CBV values from the 10 second stimulation epochs. The baseline ΔR_2_* value was calculated as follows: Baseline ΔR_2_* = −1/TE ln(S_prestim_/S_0_), where S_prestim_ and S_0_ represents MR signal intensity after and before Feraheme injection. Stimulus evoked ΔR_2_* values were calculated as follows: Stimulus evoked ΔR_2_* = −1/TE ln(S_stim_/S_prestim_), where S_stim_ and S_prestim_ are the MR signal intensities during and before stimulation, respectively. Cerebral blood volume changes were calculated by dividing stimulus-evoked ΔR_2_* by baseline ΔR_2_* values; because S_prestim_ is used to calculate baseline ΔR_2_* for each stimulation session, this corrects for any potential baseline drift. Statistical comparisons of DBS frequency effects on ΔCBV for each ROI were conducted using Graphpad Prism software (San Diego, CA). Two-tailed, one-way repeated measures ANOVA tests were conducted with Tukey post-hoc analyses. Statistical significance was set at *p* ≤ 0.05.

### Functional Connectivity Data Processing and Statistical Analyses

Functional scans were preprocessed using the Analysis of Functional NeuroImages software suite (AFNI v2011-12-21-1014). The workflow included discarding the first 20 volumes, slice-timing correction, motion correction, alignment to a pre-existing high-resolution T_2_-weighted template, spatial smoothing (Gaussian kernel FWHM = 1.5 mm), low-pass filtering (0.001 Hz), and regression of whole brain signal and the six motion parameters. The number of volumes discarded was increased from the traditional number (approximately 3–10) in order to ensure DBS-related changes from the initial stimulation were minimized. Furthermore, warping in the alignment procedure was limited to shifts and rotations to avoid unnecessary shearing and scaling of brain regions with signal drop-out associated with the DBS electrode. fcMRI analyses were conducted using the temporal correlation method. Fisher-Z transformed correlation matrices were generated using the average functional time series extracted for each region-of-interest (ROI) in the template atlas. Left (LH) and right hemispheres (RH; ipsilateral to DBS electrode) ROIs were analyzed separately yielding correlation matrices detailing within and between hemisphere connectivity. The ROIs were further separated into two putative networks, a NAc-DBS network consisting of regions with robust DBS-evoked responses, as well as the ventral tegmental area (due to its highly established involvement in NAc circuitry)^3^ versus Other, a network made up of 30 miscellaneous brain regions. The NAc-DBS network included the following 15 regions: prelimbic cortex (PLC), infralimbic cortex (ILC), orbitofrontal cortex (OFC), cingulate cortex (CC), insula, nucleus accumbens (NAc), anterior striatum (AS), ventral pallidum (vPall), septum (Sept), lateral hypothalamus (lHyp), amygdala (Amyg), bed nucleus of the stria terminalis (BNST), mediodorsal thalamus (MDT), ventral hippocampus (vHipp), and the ventral tegmental area (VTA). Repeated measures analyses of variance (MATLAB, rANOVA) were implemented in order to test the effects of the stimulation paradigm (Pre-DBS Rest, DBS, Post-DBS Rest) and the interaction with mean connectivity responses within and between hemispheres (RH, LH, RH ↔ LH) across animals (*n* = 7). P-values were assessed using the more conservative lower-bound estimate (p_lb_) in order to correct for potential violations in symmetry.

Individual connections, or pair-wise correlations (i.e. NAc ↔ OFC, etc), were further evaluated in the context of the stimulation paradigm and relative direction of effect (Connectivity versus Condition). Direction of effect was determined by fitting each significant (rANOVA, *p* ≤ 0.05 uncorrected) set of pair-wise connections with a 2^nd^ -order polynomial. The resulting sign of the quadratic term was used to categorize connections as either enhanced (increased correlation) or suppressed (increased anti-correlation). Pair-wise connections with weak modulation (|Δ_Z-corr._| < 0.10) were ignored. Finally, connections were grouped based on network classifications (sensorimotor, executive, limbic, and between network connections) and then visualized on coronal and volumetric representations of the rat template brain.

### Optogenetic Constructs

For optogenetic experiments, neuronal opsin expression was achieved using adeno-associated viral vectors (AAV, serotype 5), encoding either a humanized variant of ChannelRhodopsin-2 (hChR2; H134R) fused to an enhanced yellow fluorescent protein (EYFP), or EYFP alone. Both constructs were placed under the calcium-calmodulin kinase IIα (CaMKIIα) promoter to target striatal neurons. Viral titers were approximately 5.0 × 10^12^ viral genome/ml. All viruses were obtained from the Vector Core at the University of North Carolina at Chapel Hill.

### Histology

Following scan procedures, rats were deeply anesthetized with a 1–2 ml cocktail of pentobarbital sodium and phenytoin sodium (Euthasol) and transcardially perfused with saline followed by 10% formalin. Extracted brains were stored overnight in 10% formalin and transferred to a 30% sucrose solution (in DI water) for 2–3 days, until brains sunk to bottom of storage bottles. Brains were cut to 40 μm thick sections on a freezing microtome and mounted on glass slides for fluorescent imaging. Vectashield mounting medium with DAPI stain (Vector laboratories, Item # H-1200) was used to provide a cell body counterstain. Slides were imaged using a Zeiss 780 confocal microscope.

## Results

Electrode targeting accuracy within the NAc was verified using T_2_-weighted anatomical scans (Fig. 1B). Given the possibility of current spread to the shell region during DBS, we did not differentiate between these anatomical subdivisions in this study.

To evaluate the downstream neural circuitry modulated by NAc-DBS at 130 Hz (300 μA), CBV functional activation maps were generated using an evoked-fMRI acute stimulation paradigm (Fig. 2). These maps revealed positive CBV responses to NAc-DBS in many brain regions with known executive and/or limbic functions; these included: amygdala, infralimbic cortex, lateral hypothalamus, NAc, prelimbic cortex, septum, ventral hippocampus, and ventral pallidum. Positive CBV changes observed along the electrode tract were time-locked to the stimulation period, and not present in DBS-fMRI studies conducted with the same electrode at different targets (e.g., thalamus)^14^, strongly suggesting a neural origin. Minor CBV decreases were also detected in a small subregion of the dorsolateral striatum. Interestingly, although each of these regions has known anatomical connectivity with the NAc (in many cases as nonreciprocal glutamatergic inputs to the NAc)^32^, anatomical connectivity alone was insufficient to predict responsive brain regions. For example, the ventral tegmental area, which is densely and reciprocally connected to the NAc^33^,^34^, did not show detectable CBV modulation by 130 Hz NAc-DBS (see Fig. 2 and Supplemental Figure S3).

**Figure 3:**
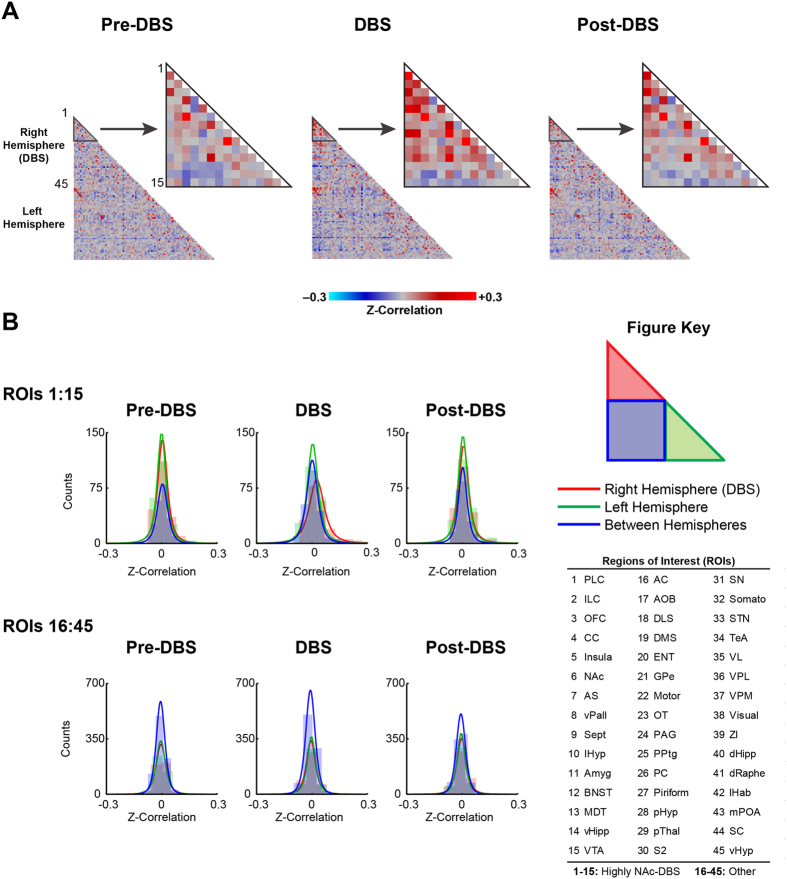
fcMRI modulation by 130 Hz NAc-DBS. **(A)** Mean correlation matrices (*n* = 7) for each stimulus condition (Pre-DBS, DBS, Post-DBS) using 45 regions-of-interest (ROIs, see Figure Key). ROIs were chosen *a priori*, with reference to anatomical regions described in a standard rat brain atlas^35^. Note the presence of between-hemispheric regional connectivity (displayed as a red diagonal line) in all matrices. **(B)** Histograms and t-distribution fits for each stimulus condition, network (NAc-DBS [ROIs #s 1–15] and Other [ROIs #’s 16–45]) and connectivity grouping (within and between hemispheres) using correlation measures from the average correlation matrices. Abbreviations: **PLC**: Prelimbic Cortex; **ILC**: Infralimbic Cortex; **OFC**: Orbitofrontal Cortex; **CC**: Cingulate Cortex; **Insula**: Insular Cortex; **NAc**: Nucleus Accumbens; **AS**; Anterior Striatum; **vPAll**: Ventral Pallidum; **Sept**: Septum; **lHyp**: Lateral Hypothalamus; **Amyg**: Amygdala; **BNST**: Bed Nucleus of the Stria Terminalis; **MDT**: Mediodorsal Thalamus; **vHipp**: Ventral Hippocampus; **VTA**: Ventral Tegmental Area; **AC**: Auditory Cortex; **AOB**: Accessory Olfactory Bulb; **DLS**: Dorsolateral Striatum; **DMS**: Dorsomedial Striatum; **ENT**: Entorhinal Cortex; **GPe**: External Globus Pallidus; **Motor**: Motor Cortex (Primary and Secondary); **OT**: Olfactory Tubercle; **PAG**: Periaqueductal Grey; **PPTg**: Pedunculopontine Tegmental Nucleus; **PC**: Parietal Cortex; **Piriform**: Piriform Cortex; **pHyp**: Posterior Hypothalamus; **pThal**: Posterior Thalamus; **S2**: Secondary Somatosensory Cortex; **SN**: Substantia Nigra; **Somato**: Primary Somatosensory Cortex; **STN**: Subthalamic Nucleus; **TeA:** Temporal Association Cortex; **VL**: Ventrolateral Thalamus; **VPL**: Ventral Posterolateral Thalamus; **Visual**: Visual Cortex (Primary and Secondary); **ZI**: Zona Incerta; **dHipp:** Dorsal Hippocampus; **dRaphe**: Dorsal Raphe Nucleus; **lHab**: Lateral Habenula; **mPOA**: Medial Preoptic Area; **SC**: Superior Colliculus; **vHyp**: Ventral Hypothalamus.

Next, we undertook fcMRI experiments to study the effects of NAc-DBS over longer time periods (minutes). Previous electrophysiological and neuroimaging studies have provided evidence of network synchrony modulation by high frequency NAc-DBS^15^,^35^. Exploiting the capability of fcMRI to measure functional connectivity on a whole-brain scale, we mapped global network changes induced by NAc-DBS at 130 Hz (300 μA). Mean correlation matrices were generated for each stimulus condition (Pre-DBS, DBS, Post-DBS) across 90 anatomically-defined ROIs (45 ROI’s per brain hemisphere) (Fig. 3A). These ROIs were grouped into two pre-defined networks, based on our evoked fMRI findings (see Methods; NAc-DBS [#s 1–15] and Other [#’s 16–45]). A complete listing of ROIs and network groupings is provided in the Fig. 3 key and associated figure caption. For the Pre-DBS condition, bilateral connectivity was evident between homologous ROIs, and for certain structures appeared more robust (i.e. PLC, ILC, OFC, CC, Insula, Motor, etc). The presence of bilateral functional connectivity, as displayed in our datasets, represents a key feature of fcMRI^36^. Except for a small number of ROIs (i.e. NAc ↔ OFC), within hemisphere connectivity was overall low in the Pre-DBS condition, possibly due to the short sampling period (5 mins per trial). However, during NAc-DBS of the RH NAc, within RH connectivity (i.e. ipsilateral to the DBS site) increased between many of the “NAc-DBS related network” ROIs, whereas there was little apparent modulation in the “Other network”. Between NAc-DBS network connectivity also appeared slightly modulated in the DBS condition but for a smaller number of ROIs (i.e. PLC, ILC). Post-DBS connectivity for the DBS-NAc network was similar, or perhaps intermittent to the pre-DBS / DBS conditions.

Next, histograms were generated (Fig. 3B) and fit as t-distributions for each stimulus condition, network, and connectivity grouping (within and between hemispheres) using average measures from the connectivity matrices. Consistent with the previous observations, NAc-DBS network connectivity within the RH was the only distribution to show enhanced positive correlations during DBS stimulation of the RH NAc. Repeated measures analyses of variance (rANOVA) were used to statistically validate these results across animals. For the NAc-DBS network, the stimulus condition (Pre-DBS, DBS, Post-DBS) and interaction between condition and connectivity (RH, LH, RH ↔ LH) were significant (p_lb_ ≤ 0.05); *Condition* F(2,41) = 7.45, p_lb_ = 0.014 and Condition*Connectivity F(4,41) = 4.15, p_lb_ = 0.033.

**Figure 4:**
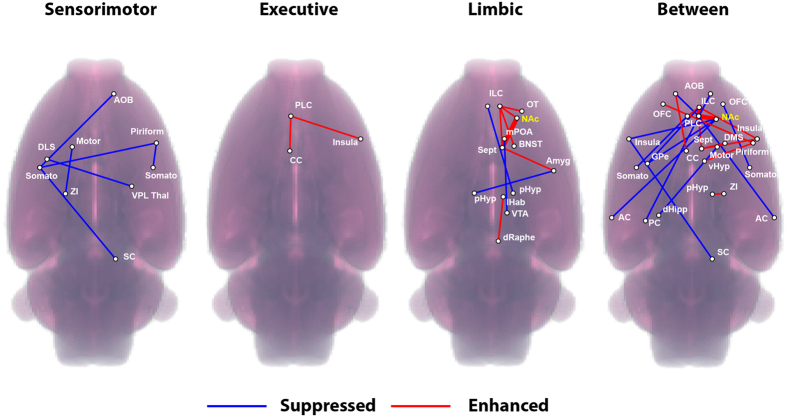
Network-level visualization of pair-wise fcMRI modulations during 130 Hz NAc-DBS. Significant (rANOVA, *p* ≤ 0.05 uncorrected, Δ_Z-Corr_ > 0.10) enhanced (red) or suppressed (blue) individual pair-wise connections grouped by functionally-defined networks (Sensorimotor, Executive, Limbic, and Between Network Connections). Thick red lines represent Δ_Z-Corr_ > 0.20 at R-NAc ↔ R-Sept, R-PLC and R-ILC ↔ R-PLC.

Visualization of significantly (rANOVA, *p* < 0.05 uncorrected, Δ_Z-Corr_ > 0.10) enhanced (increased correlation) or suppressed (decreased correlation or increased anti-correlation) individual pair-wise connections grouped by functionally-defined networks (Sensorimotor, Executive, Limbic, and Between Network Connections) paralleled the above observations and revealed additional information that was not readily apparent in the original classification of the data (Fig. 4; blue-suppressed, red-enhanced). Enhancement was primarily restricted to connections between RH limbic structures, however consistent suppression was also evident, mainly within the sensorimotor network and between limbic and motor regions. The sensorimotor network showed no significant enhancement, and moreover, the executive network was devoid of suppressed connections. Enhancement was relatively robust (Fig. 4, Δ_Z-Corr_ > 0.20 thick red lines) for the following connections; R-NAc ↔ R-Sept, R-PLC and R-ILC ↔ R-PLC. Additional analyses of fcMRI data are provided in Supplemental Figures S5 and 6.

**Figure 5:**
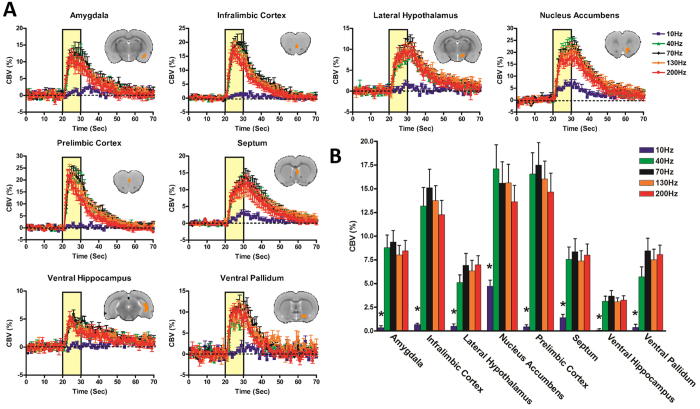
Temporal dynamics **(A)** and amplitudes **(B)** of CBV responses to NAc-DBS across five stimulation frequencies (10, 40, 70, 130, 200 Hz; *n* = 8 per frequency), demonstrating that CBV responses to NAc-DBS were largely stimulation frequency-insensitive. All subjects were scanned with 500 μA DBS, except one subject with 600 μA. CBV responses are expressed as a percent change from pre-stimulation baseline values. Amplitudes were calculated as mean percent CBV changes during stimulation epochs (10 seconds; scan frames 21–30). Anatomically-defined ROIs are highlighted as figure inserts in **5A** (Single slice shown; note that many ROIs encompassed multiple slices). *p ≤ 0.05 for 10 Hz compared to all other frequencies (no other statistically significant comparisons). Datapoints are presented as mean ± SEM.

To investigate the frequency-dependency of CBV responses to NAc-DBS, we next conducted additional evoked-fMRI experiments with five DBS frequencies (10, 40, 70, 130 and 200 Hz). To achieve a more robust measurement of the frequency effects and circumvent the issue of low DBS-fMRI sensitivity at low stimulus amplitude this experiment was conducted using a slightly higher current intensity at 500 μA^37^. The results generated CBV response patterns that were qualitatively similar, yet more robust responses than 300 μA (see Supplemental Figure S4). This current remains to be lower than in many previously reported DBS-fMRI studies^12^,^14^,^37^,^38^, and we thus do not expect significant off-target effects. To further reduce bias towards characterizing off-target areas with this stronger current amplitude, we only evaluated anatomical ROIs that were significantly modulated with 300 μA stimulation (at 130 Hz; Fig. 2). This ROI-based analysis included two complementary measures of regional activity modulation: CBV time-courses and amplitudes; both are quantitative measures of percent CBV changes. CBV time-courses generated from 10 Hz NAc-DBS resulted in weak or no change in CBV (though see Supplemental Figure S4 demonstrating sparse CBV increases in and surrounding the NAc), whereas all other frequencies resulted in region-specific CBV increases of similar amplitudes and rise/decay kinetics (Fig. 5A). The absence of distal CBV responses with 10 Hz NAc-DBS is remarkable, as DBS at this stimulation frequency has been shown to generate robust downstream fMRI signals at other target locations (e.g., the ventroposteromedial thalamus)^14^. In general, NAc-DBS induced sharp rises in CBV that returned to baseline values by the end of the test period (i.e., with 40 s of recovery). The largest CBV increases were detected in the NAc, infralimbic, and prelimbic cortices, each displaying maximal increases in CBV ~20% above baseline values. Among all regions showing significant and detectable responses, the ventral hippocampus displayed perhaps the weakest, with a maximal CBV increase of just over ~5% from baseline. Interestingly, the duration to peak CBV amplitude varied in a region-specific manner. For example, CBV continued to increase in the lateral hypothalamus and septum for the duration of the stimulation period, while CBV values peaked and decayed more rapidly in prefrontal cortex. Quantitative comparisons of CBV amplitude modulation by NAc-DBS at varying frequencies are shown in Fig. 5B. One-way ANOVAs revealed a significant main effect of NAc-DBS frequency on the amplitude of CBV change for all regions examined. Remarkably, post-hoc testing revealed that while 40–200 Hz NAc-DBS significantly increased CBV compared to the 10 Hz condition, no other significant frequency-dependent effects of NAc-DBS on CBV amplitude were noted. That is, increasing the frequency of NAc-DBS from 40 to 200 Hz, a five-fold difference, did not further modulate the functional circuit responses in the structures that we investigated. Critically, evoked responses were generally consistent across multiple stimulation trials, arguing against the possibility of stimulation-induced neural damage.

**Figure 6:**
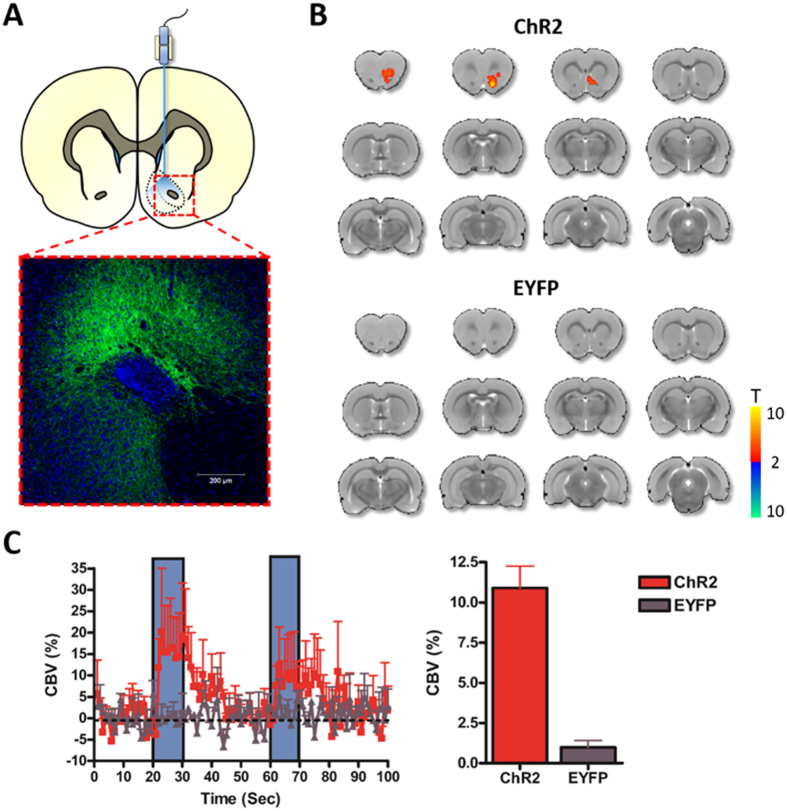
Optogenetic stimulation at NAc evokes local CBV increases. **(A)** Schematic of optogenetic stimulation at NAc (top), representative confocal image confirming ChR2 expression (green) in the NAc (bottom). Counterstain is DAPI (blue). ChR2 expression appeared strongest in the core subregion of the NAc. **(B)** Functional CBV responses induced by 40 Hz optogenetic stimulation at NAc in animals expressing ChR2 or EYFP (AAV5 using the CaMKIIα promoter; *n* = 4 and 2, respectively). Note that optogenetic stimulation of the NAc resulted in CBV increases locally within the stimulated region, with no detected downstream responses. No responses were observed in EYFP subjects. Anteroposterior slice coordinates are as described for Fig. 2. Temporal CBV dynamics **(C)** and amplitudes **(D)** within the NAc during local optogenetic stimulation. Stimulation-evoked CBV amplitude changes were calculated as described in Methods.

In addition to electrical stimulation, optogenetic tools are being increasingly exploited for mapping functional connectivity among neural circuits. Several studies have also employed optogenetic stimulation to elucidate potential mechanisms of therapeutic DBS action, including at the nucleus accumbens target^39^. In experiments reported here, we explored the possibility of using optogenetic NAc stimulation to induce detectable downstream fMRI signals. This pilot experiment was performed in rats that received viral injections of an adeno-associated virus encoding ChR2 under the neuron-specific CaMKIIα promoter. A representative example of virally-mediated ChR2 expression in NAc neurons is provided in Fig. 6A. Optogenetic stimulation at 40 Hz evoked a local positive CBV signal in the NAc, with no significant CBV modulation observed outside the target region (Fig. 6B,C). Ongoing studies in our laboratory using a completely identical experimental setup and analysis pipeline have revealed robust optogenetic-induced responses in downstream areas far from the stimulation site. Thus, we are confident that our findings do not reflect a technical failure to manipulate NAc neurons optogenetically.

A recent study has demonstrated hemodynamic changes following blue light laser stimulation (50% duty cycle) in naïve rat brain, possibly due to a laser heating artifact^40^. To evaluate the possibility of such an artifact as the source of the observed optogenetic response at the NAc, we repeated our optogenetic-fMRI experiments in control rats expressing an inert EYFP fluorophore in the NAc. In these animals, no significant CBV modulation by NAc-DBS was observed (Fig. 6B,C). It is worth noting that our laser pulse duration was substantially shorter than that previously reported to induce laser artifacts, likely explaining the absence of such a response caused by laser heating. Local heating artifacts in optogenetic-fMRI experiments have since been examined in greater detail, including suggested control experiments to rule out such artifacts as causal in fMRI signal generation^41^.

## Discussion

In this study, we performed multimodal fMRI procedures to identify neural circuitry modulated by NAc-DBS in a healthy rat model. Evoked fMRI with electrical DBS uncovered a broad range of cortical and subcortical regions displaying stimulation-induced CBV increases, including prefrontal cortex, lateral hypothalamus, amygdala, septum, and ventral hippocampus. fcMRI provided corroborative evidence of robust network modulation by NAc-DBS, including suppression of sensorimotor and enhancement of executive and limbic network connectivity. Lastly, we performed opto-fMRI with selective and direct stimulation of NAc neurons, showing time-locked CBV increases exclusively localized in the NAc during 40 Hz optical stimulation.

Although high frequency DBS is generally reported to locally silence neuronal activity^42^,^43^, we noted only CBV increases in the target region (i.e., the NAc) across all tested stimulation frequencies. This finding is consistent with a number of human neuroimaging studies reporting increases in hemodynamic responses or glucose metabolism in the subthalamic nucleus (STN) during STN-DBS^7^. Notably, each of these studies (including our own) uses indirect measures of neuronal activity, and thus this seeming paradox may possibly be explained by an uncoupling of hemodynamic responses from neuronal spiking during high frequency stimulation. DBS may induce both depolarization blockade and augmented presynaptic activity, either of which may potentially increase local metabolic demand in the absence of somatic spiking^44^. In this context, it is also interesting to note several reports of local increases in c-fos expression following DBS of the NAc, STN, or pedunculopontine tegmental nucleus in rats^45^,^46^,^47^. Future studies may determine the nature and extent of this possible uncoupling, which has strong implications for the interpretations of neuronal activity based on indirect experimental measures such as fMRI or immediate early gene expression.

Complementing our evoked-fMRI findings of direct limbic circuit modulation by NAc-DBS, fcMRI measurements revealed robust enhancements in functional connectivity between many of these same regions. More generally, limbic and executive network connections were primarily enhanced but those in the sensorimotor network were largely suppressed. These findings suggest that NAc-DBS modulates multiple functional network domains, spanning the entire brain. Although these functional connectivity maps alone do not allow for causal inferences relating to the therapeutic mechanism of NAc-DBS, they should provide an invaluable resource for the generation of future, hypothesis-driven DBS studies.

A central tenet of DBS therapy is its dependence upon high stimulation frequencies. One widely held hypothesis of DBS action posits that high frequency stimulation masks pathological endogenous circuit activity, thus creating an “informational lesion” at the DBS target (although other mechanisms have also been posited)^48^,^49^,^50^. We were thus surprised to find that the functional activation profile obtained by NAc-DBS in rats was remarkably insensitive to a wide range of stimulation frequencies; indeed, 40 Hz stimulation modulated CBV across all tested areas in a manner quantitatively similar to all higher frequencies tested, including 200 Hz. Although we used a healthy rodent model, precluding behavioral tests of DBS efficacy, our evoked-fMRI results highlight strong parallels in circuit modulation by either moderate or high frequency stimulation of the NAc. Further, a recent preclinical study of NAc-DBS for cocaine addiction reported similar attenuation of reinstated drug-seeking behavior at 20 or 160 Hz, suggesting that some behavioral effects of NAc-DBS may also be frequency-insensitive^17^. The frequency sensitivity of symptom amelioration by NAc-DBS would be interesting to study in the clinical setting, particularly in light of the possibility of prolonging the battery life of DBS pulse generators using lower frequency stimulation protocols.

In this study, we have also provided preliminary data concerning the fMRI signal pattern generated by optogenetic stimulation of the NAc in a small number of subjects. We were surprised to discover that, in our setup, optogenetic NAc stimulation evoked detectable CBV increases solely within the NAc, whereas identical parameters used at other target regions revealed significant downstream responses. The lack of distal responses in the present experiment is particularly perplexing in that neural activity within the target region itself was clearly and robustly modulated by optogenetic stimulation. The reason for such large differences in circuit modulation patterns between optogenetic and electrical NAc stimulation is unclear, although several possibilities exist. First, stimulus intensity differed between optogenetic and electrical DBS in this study, which may contribute to the differing activation patterns. Second, although we have assumed that both electrical and optogenetic stimulation delivered at 40 Hz would activate (rather than suppress) neural circuits, this was not formally tested. Another possibility concerns more fundamental differences between these stimulation approaches, such as the confinement of optogenetic stimulation to the opsin-expressing neurons and their efferent fibers. However, we also cannot entirely rule out the possibility that ChR2 was expressed in a small population of non-neuronal cells. The most likely explanation for our findings, in our opinion, is that most of the circuits recruited by NAc-DBS were resulted from antidromic activation of axons or fibers of passage. Our future studies will focus on evaluating this hypothesis.

There are several limitations to the present study to consider. First, although the NAc is generally considered to be comprised of two related, yet distinct subdivisions (core and shell) each with distinct circuit features^3^,^32^. We did not distinguish between these two areas in our experiments. Indeed based on the obtained functional response profiles, both regions were likely recruited by our stimulation paradigm. The core and shell subregions are both candidate therapeutic targets for neuropsychiatric DBS, but the efficacy of each may strongly depend upon the disorder being treated. For example, NAc-DBS for OCD generally targets the core region^51^,^52^, while some preclinical studies suggest that the shell is more effective for addictive disorders^19^,^46^,^53^. Other behavioral phenotypes, such as quinpirole-induced checking behavior (a pharmacological model of OCD symptoms), are similarly influenced by NAc-DBS of the core or shell^54^. Further studies are necessary to elucidate the downstream circuitry that confers unique therapeutic properties of DBS at either NAc subdivision.

A second major limitation of our study was the usage of dexmedetomidine sedation^27^,^36^, which may alter the responsivity of neural circuits to the effects of DBS. Related to this point, we were unable to reliably achieve robust fMRI responses with current amplitudes below 300 μA (data not shown), and thus our experiments relied upon higher amplitudes than those generally used in preclinical DBS studies in awake rodents (typically 100–150 μA)^15^,^16^,^19^, although amplitudes higher than 150 μA have been reported for studies in both awake and anesthetized states^18^,^55^. We postulate that the relatively higher current amplitudes needed for DBS effects in our model may have been necessitated by the sedation state, the detection sensitivity of our fMRI measurements, or both.

A third limitation concerns the use of an acute stimulation paradigm, which was necessary in the context of our experimental MR setup. Similar OFF-ON-OFF paradigms are traditionally used in DBS-fMRI studies^11^,^12^,^13^,^56^, although they may provide a biased perspective on DBS-evoked changes in functional connectivity. A recent report by Ewing and Grace highlights the importance of studying DBS network effects under chronic stimulation conditions^15^. In that study, local field potentials (LFPs) were recorded in multiple brain regions of rats receiving continuous NAc-DBS for a period of 5 days. Some of the observed DBS-induced network changes were transient, including enhanced delta power in the mediodorsal thalamus and orbitofrontal cortex, whereas other network effects emerged over time (e.g., decreased alpha power in the mediodorsal thalamus). This work highlights the importance of studying DBS effects over more translationally-relevant timespans (days or weeks, if experimentally feasible). fcMRI, which does not require within-session baseline (“OFF”) periods, provides an ideal experimental measure for future longitudinal examinations of DBS effects across time and brain regions.

In the present study, we identified a robustly recruited network of cortical and subcortical circuits modulated by NAc-DBS, many of which are likely to be antidromically-stimulated. Remarkably, the extent of this circuit modulation was relatively frequency-insensitive, as demonstrated by our evoked-fMRI findings. Collectively, our findings should facilitate the understanding of DBS mechanisms and mapping of therapeutic circuits at this important clinical target. Future NAc-DBS fMRI studies should examine the circuit- and network-level responses to therapeutic stimulation in neuropsychiatric disease models, wherein pathology-specific circuit disruptions (and putative amelioration by DBS) may be explored.

## Additional Information

**How to cite this article**: Albaugh, D. L. *et al*. Functional Magnetic Resonance Imaging of Electrical and Optogenetic Deep Brain Stimulation at the Rat Nucleus Accumbens. *Sci. Rep*. **6**, 31613; doi: 10.1038/srep31613 (2016).

## Supplementary Material

Supplementary Information
