# Supplementary material for: Functional Magnetic Resonance Imaging of Electrical and Optogenetic Deep Brain Stimulation at the Rat Nucleus Accumbens
Source: Sci Rep. 2016 Sep 7;6:31613. doi: 10.1038/srep31613 (PMC5013271; doi:10.1038/srep31613)
Supplement: Supplementary Information [file srep31613-s1.pdf]

# Supplementary Materials

# Functional Magnetic Resonance Imaging of Deep Brain Stimulation at the Rat Nucleus Accumbens

Daniel L. Albaugh[1-3], Andrew Salzwedel[2,6,7], Nathalie Van Den Berge[1-2,8], Wei Gao*[2,6,7], Garret D. Stuber*[3-4], and Yen-Yu Ian Shih*[1-3,5]

*Co-corresponding authors.

[1]Department of Neurology, University of North Carolina, Chapel Hill, NC, USA, 27599

[2]Biomedical Research Imaging Center, University of North Carolina, Chapel Hill, NC, USA, 27599

[3]Curriculum in Neurobiology, University of North Carolina, Chapel Hill, NC, USA, 27599

[4]Department of Psychiatry, University of North Carolina, Chapel Hill, NC, USA, 27599

[5]Department of Biomedical Engineering, University of North Carolina, Chapel Hill, NC, USA, 27599

[6]Department of Radiology, University of North Carolina, Chapel Hill, NC, USA, 27599

[7]Biomedical Imaging Research Institute, Department of Biomedical Sciences and Imaging, Cedars-Sinai Medical Center, Los Angeles, CA, USA, 90048

[8]Medical Image and Signal Processing Group, Ghent University, Ghent, Belgium, 9000

## Supplemental Figures

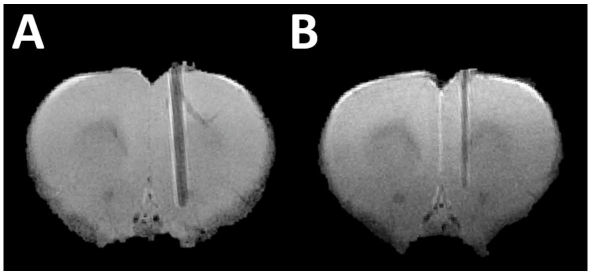


**Figure S1**. Representative $T_2$-weighted anatomical images displaying electrode placement in the NAc **(A)** and optic fiber placement above the NAc **(B)**. Note the minimal electrode artifact with the tungsten microwire electrode.

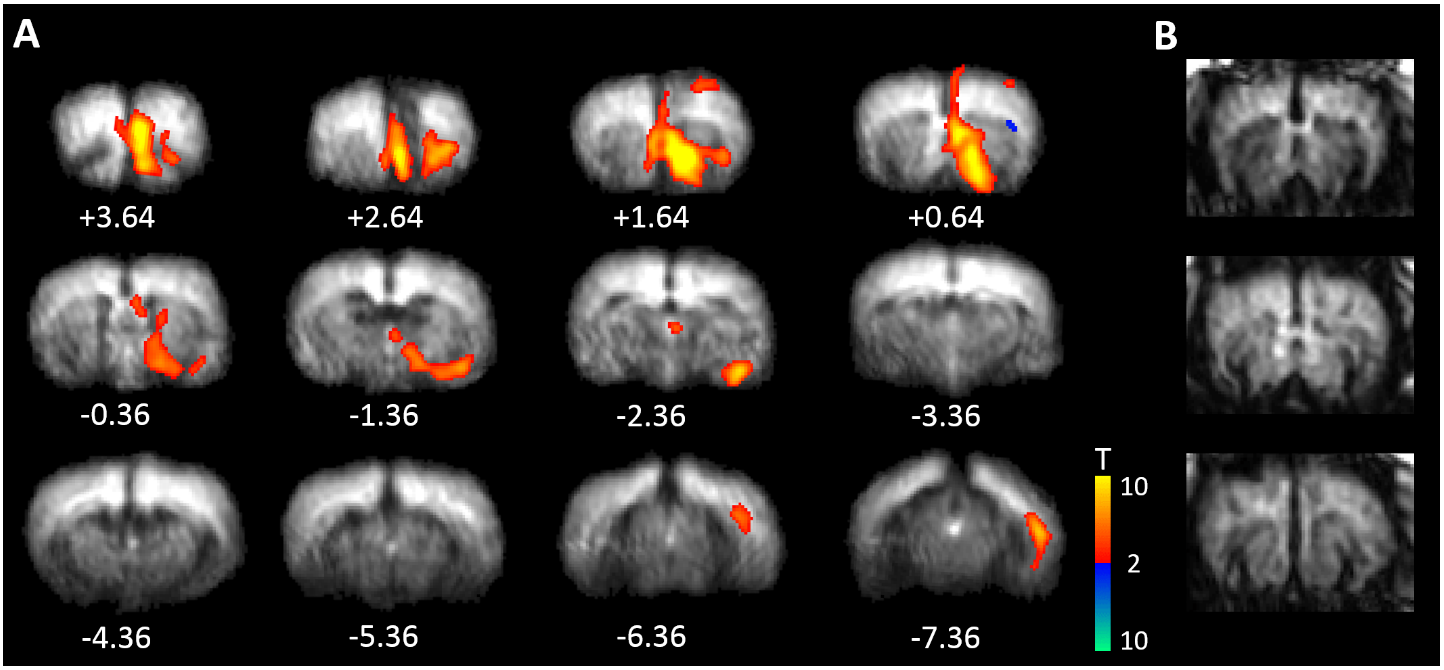


**Figure S2**. NAc-DBS-evoked functional activation maps (130 Hz; 300 μA) overlaying on group-averaged EPI images (*n* = 5), presented on group-averaged EPI data **(A)**. For comparison, the template-overlaid images are presented in **Figure 2B**. A sample of raw, unaveraged EPI images from 3 subjects is provided in **(B)**. As we achieved robust evoked-fMRI responses in all animals, no data was discarded, including subject EPI data displaying artifacts. Additional details regarding these images are located in the **Figure 2** caption.

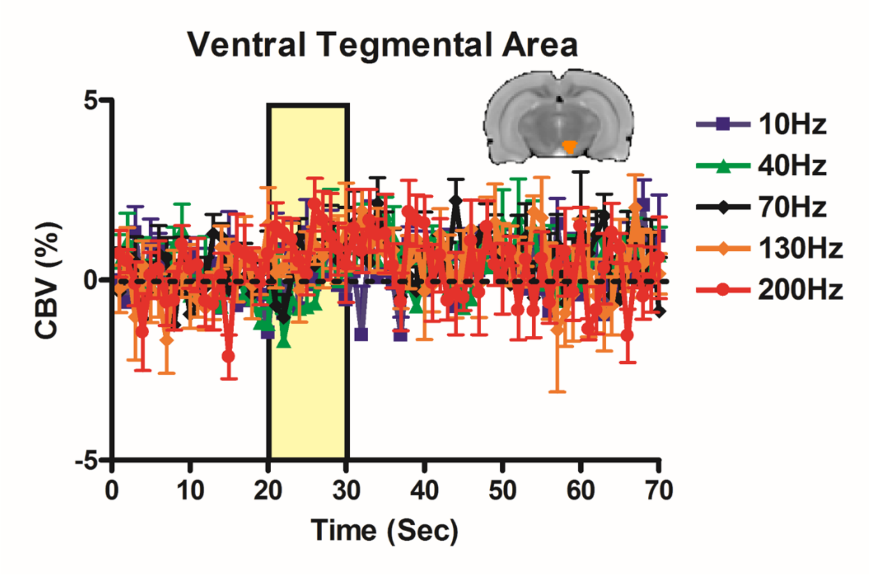


**Figure S3**. Temporal dynamics of VTA CBV responses to NAc-DBS across five stimulation frequencies (10, 40, 70, 130, 200 Hz; *n* = 8 per frequency) showing a lack of detectable evoked responses. All subjects were scanned with 500 μA DBS, except one subject with 600 μA. Additional details are provided in the **Figure 5** caption.

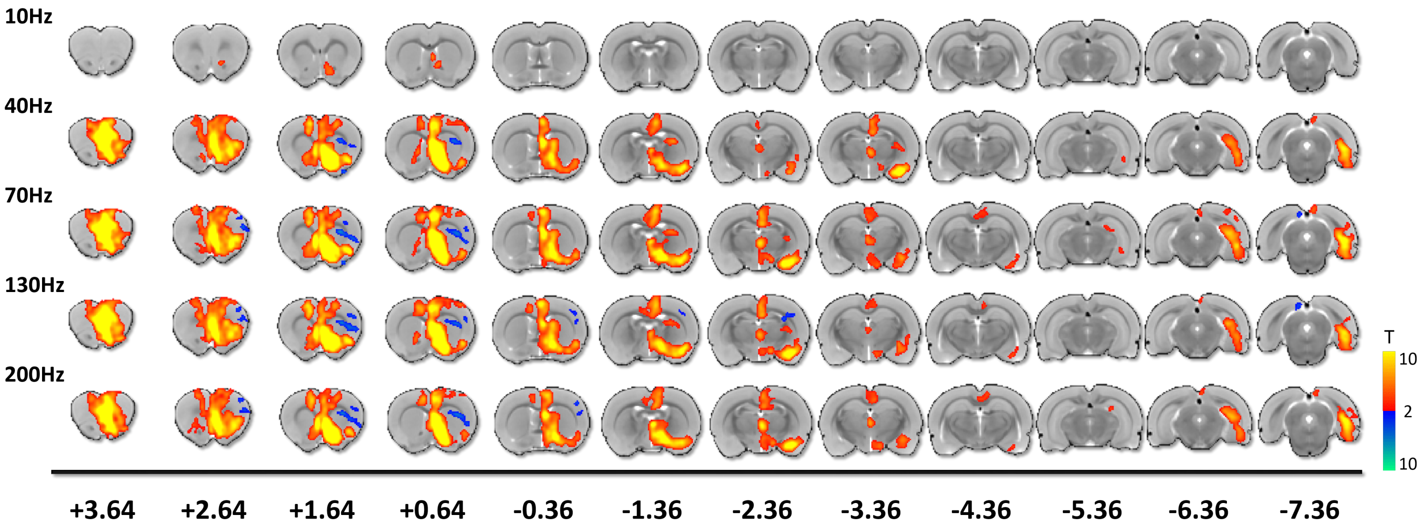


**Figure S4**. Functional activation maps of CBV modulation by NAc-DBS at 10, 40, 70, 200, and 400 Hz (500 μA; except for one subject with 600 μA) (*n* = 8 per frequency). NAc-DBS delivered at 10 Hz resulted in sparse CBV increases within and around the NAc. DBS delivered at all other tested frequencies resulted in large-scale CBV modulation in both cortical and subcortical areas, which further were remarkably similar in spatial patterning across DBS frequencies (see also **Figure 5**). Additional details regarding these images are located in the **Figure 2** caption. Slice locations (reference to the Bregma in mm) are labeled at the bottom of the figure.

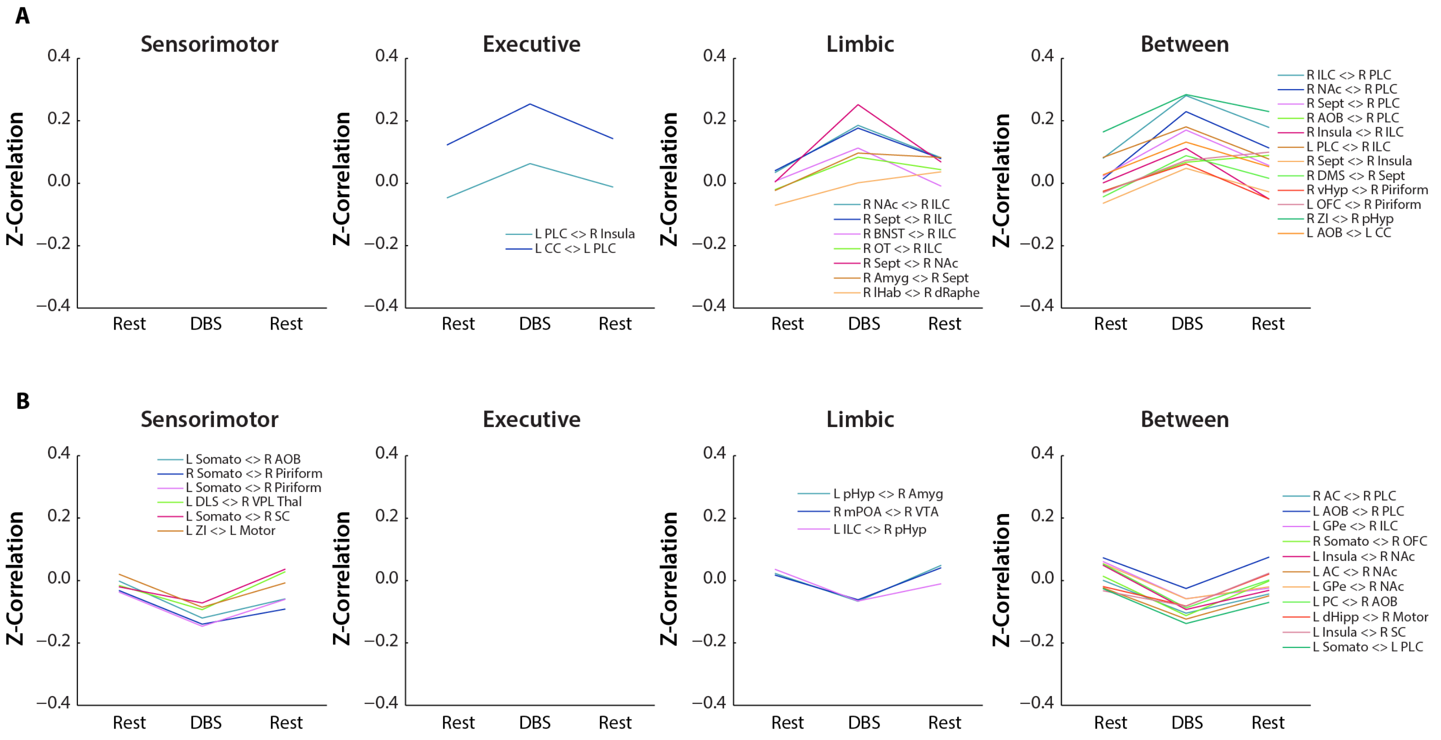


**Figure S5**. Network-level visualization of pair-wise fcMRI modulations during 130 Hz NAc-DBS. Significant (rANOVA, $p \leq 0.05$ uncorrected, ΔZ-Corr > 0.10) individual pair-wise connections grouped by functionally-defined network (Sensorimotor, Executive, Limbic, and Between Network Connections) and plotted as Z-Correlation vs Stimulus Condition (Pre-DBS, DBS, Post-DBS). Pathways with enhanced connectivity are shown in **(A)** and suppressed connectivity are shown in **(B)**. Individual lines represent group means ($n = 7$).

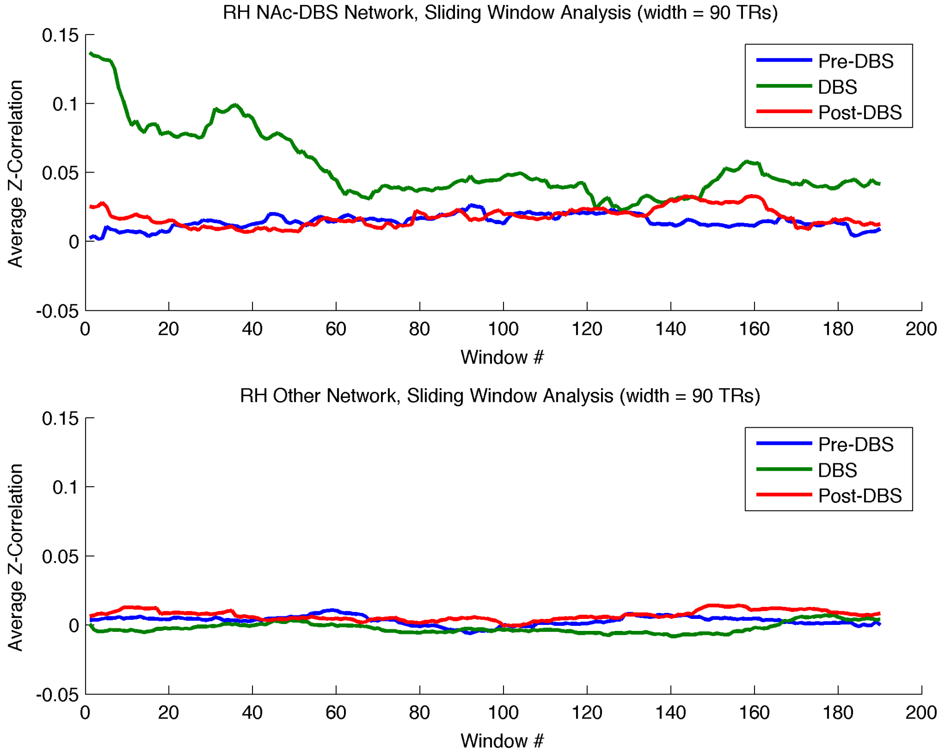


**Figure S6**. Sliding window analysis (90 s width; i.e., 1-90 s, 2-91 s…) of averaged z-correlations during the 130 Hz DBS stimulation fcMRI scan period. This analysis was conducted for ROIs within the right hemisphere (RH), ipsilateral to the stimulation site. As described in the Methods, ROIs were separated into two separate networks: the NAc-DBS Network, and Other Network. Note that, for the NAc-DBS Network (top traces), average z-correlations (i.e., connectivity strength) generally remained higher for the DBS period compared to Pre/Post-DBS periods, and were highest at the onset of stimulation. However, no significant differences were detected between the first- and second-half of the data (paired t-test; $p > 0.05$). For the Other Network (bottom traces), there were no observable differences in connectivity strength between the DBS and Pre/Post periods.
